# White-light-induced synthesis of injectable alginate-based composite hydrogels for rapid hemostasis

**DOI:** 10.1186/s40779-023-00483-7

**Published:** 2023-10-17

**Authors:** Meng-De Zhang, Xing Huang, Zhao Li, Wei Song, Yi Kong, Chao Zhang, Li-Ting Liang, Yu-Yan Huang, Ya-Xin Tan, Yu Feng, Qing-Hua Liu, Yu-Xia Zhao, Xiao-Bing Fu, Sha Huang

**Affiliations:** 1 Research Center for Tissue Repair and Regeneration Affiliated to the Medical Innovation Research Department, PLA General Hospital and PLA Medical College, Beijing, 100853 China; 2 Key Laboratory of Photochemical Conversion and Optoelectronic Materials, Technical Institute of Physics and Chemistry, Chinese Academy of Sciences, Beijing, 100190 China

**Keywords:** Photoinitiator, Photopolymerization, Alginate, Hydrogel, Hemostasis

Dear Editor,

Timely and effective hemostasis is of great significance for reducing body damage and mortality of patients [1]. Alginate is generally considered to be an excellent hemostatic polymer-based biomaterial and has been approved by the Food and Drug Administration as Generally Recognized as Safe [2]. However, the violent crosslinking reaction and unstable structure at the wound site limit its clinical applications. Hence, we report a biocompatible and injectable composite hydrogel methacrylate alginate (Alg-AEMA)-based Eosin Y/N-phenylglycine (NPG)-initiated composite hydrogel (AEC) composed of photocrosslinkable alginate, viscosity modifiers and novel white light photoinitiator, namely Eosin Y/NPG system, for instant hemorrhage control.

We first investigated the photoinitiators and corresponding light sources for Alg-AEMA photopolymerization. White light was considered non-phototoxic compared with typically used short wavelength light for photopolymerization (Additional file 1: Fig. S1a, b). However, white light photoinitiators, such as tris-bipyridyl ruthenium hexahydrate (Ru)/sodium persulfate (SPS) system, Eosin Y/triethylamine/N-vinyrrolidone (NVP) system, suffered shortcomings such as low crosslinking efficiency [3]. We reported Eosin Y/NPG as a highly efficient photoinitiator system for the preparation of hydrogels in the first instance (Fig. 1a). Upon the irradiation of the white light emitting diode (emission spectrum showed in Fig. 1b), Eosin Y (absorption spectrum showed in Fig. 1b) can be excited from the ground state to the triplet state and extract hydrogen protons from NPG. Then the NPG intermediates can experience a decarboxylation process and produce aminoalkyl radicals to induce the crosslinking of Alg-AEMA (Fig. 1a) [3]. Photopolymerization kinetic study in Fig. 1c, d indicated that the initiating efficiency [represented by the double bond conversion (DBC) of poly (ethylene glycol) diacrylate 400, DBC%] increased with the increase of NPG, but the increase of Eosin Y did not obviously change the initiation efficiency. Photopolymerization kinetic study and direct contact cytotoxicity test (Fig. 1e) were used to determine the optimal concentrations of Eosin Y and NPG for biomedical applications as 0.01% (w/v) and 0.1% (w/v), respectively. Notably, Eosin Y/NPG proved to be an efficient photoinitiator system for radical polymerization of acrylic monomers compared to the commonly used Ru/SPS system and Eosin Y/triethanolamine/NVP system (Fig. 1f). The DBC of Eosin Y/NPG group exceeded 75% after approximately 120 s of irradiation while the highest DBC reached by Ru/SPS group and Eosin Y/triethanolamine/NVP group during the test were around 20% and 18%, respectively. Highly-efficient photoinitiator system and the easily available white-light source guarantee the safety and the convenience of use of the hydrogel.

**Fig. 1:**
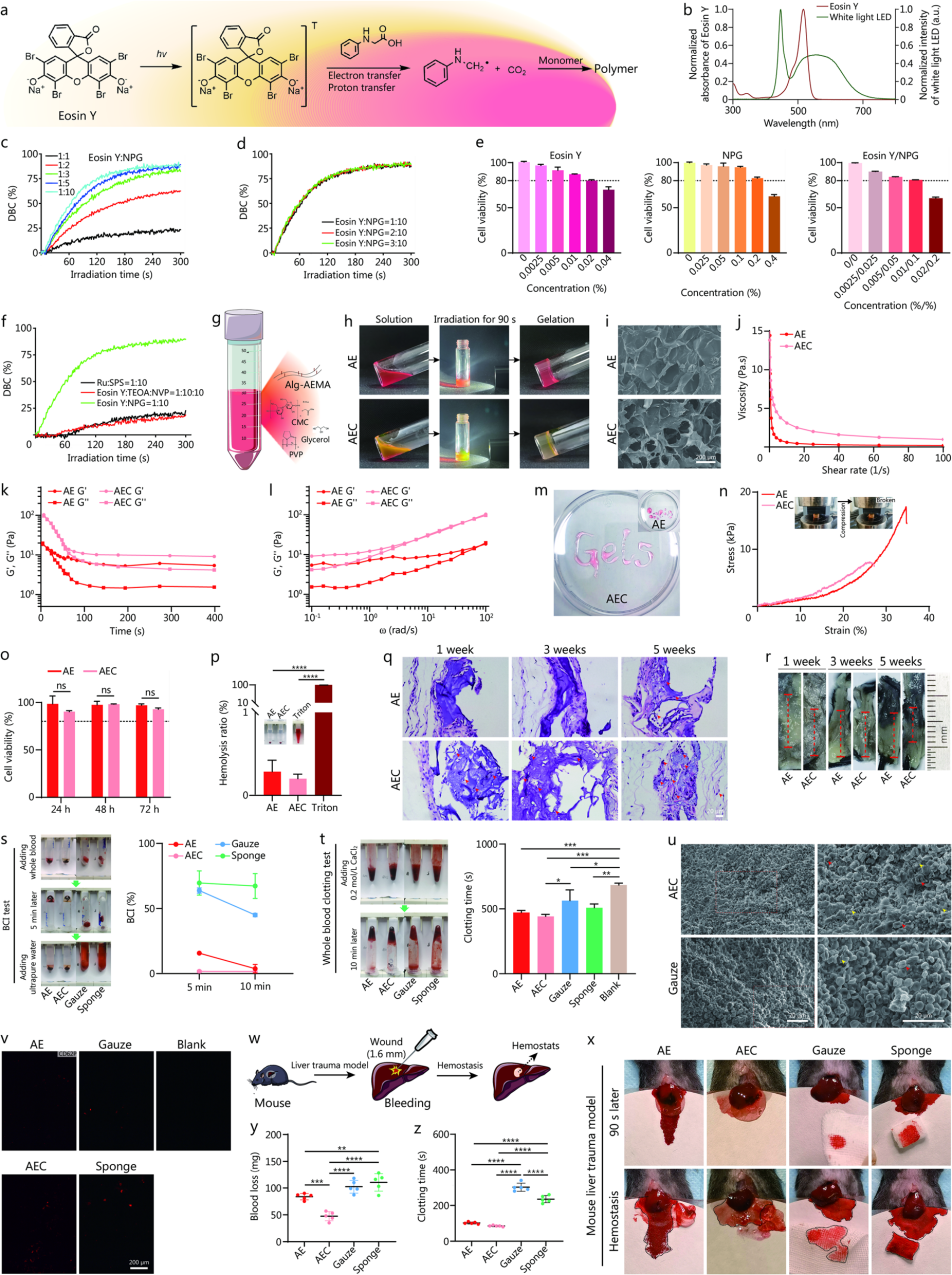
Preparation and characterization of injectable composite hydrogel with rapid hemostatic function. **a** Speculation mechanism of Eosin Y/NPG system-initiated polymerization reaction. **b** UV–visible absorption spectra of Eosin Y and emission spectrum of the white light LED. **c** and **d** Evaluation of the initiating efficiency of Eosin Y/NPG system. **e** Direct contact cytotoxicity assay of Eosin Y, NPG, and Eosin Y/NPG photoinitiator system (*n* = 3). **f** Photopolymerization kinetic experiment of various photoinitiators. **g** Schematic illustration of the composition of AEC. **h** Digital photographs of AE and AEC gelling transition upon white light irradiation (90 s). **i** SEM images of AE and AEC. Scale bar = 200 μm. **j** Viscosity versus shear rate for AE and AEC. **k** Dynamic time-sweep rheological analysis showing the gelation kinetics of AE and AEC. **l** Dynamic frequency-sweep rheological analysis of AE and AEC. **m** Macroscopic view of the injectability of AE and AEC. **n** Representative stress–strain curves of compression test. **o** Relative cell viability of NIH/3T3 fibroblasts after incubation with leaching solution of AE or AEC for 24, 48 and 72 h (*n* = 3). **p** Macroscopic view and statistical results of in vitro hemolysis assay (*n* = 3).** q** HE staining of explanted AE and AEC after 1, 3 and 5 weeks of subcutaneous implantation in mice. The red arrows represented migrated tissue cells. Scale bar = 20 μm. **r** Macroscopic view of explanted AE and AEC. The red dashed line indicated the diameter of the residual hydrogel in each group. **s** Macroscopic view of BCI test and BCI value-time curves of different samples (*n* = 3). **t** Macroscopic view of whole blood clotting test and the results showing a relatively shorter clotting time of AEC (*n* = 3). **u** SEM morphology of the clotting blood on AEC and cellulose gauze at 1000 × (left) and 2000 × (right) magnification. The yellow arrows represented fibrin network and the red arrows represented deformed red blood cells. Scale bar = 20 μm. **v** Immunofluorescence staining of CD62p (red) showing the platelet activation under the stimulation of different samples. Scale bar = 200 μm. **w** Schematic illustration of the surgical procedure of hemostasis experiments in mouse liver trauma model. **x** Photographs of the hemostatic effect of different treatments in mouse liver trauma model. Total blood loss (**y**) and clotting time (**z**) of different samples in mouse liver trauma model (*n* = 5). All statistical data are represented as mean ± SD. ^*^P < 0.05, ^**^P < 0.01, ^***^P < 0.001, ^****^P < 0.0001, One-Way analysis of variance, ANOVA, Tukey’s post hoc test. LED light emitting diode, DBC double bond conversion, NPG N-phenylglycine, AE Alg-AEMA hydrogel initiated by Eosin Y/NPG, CMC sodium carboxymethyl cellulose, PVP polyvinylpyrrolidone, Ru tris-bipyridyl ruthenium hexahydrate, SPS sodium persulfate, TEOA triethanolamine, NVP N-vinyrrolidone, AEC Alg-AEMA-based Eosin Y/NPG-initiated composite hydrogel, G' storage modulus, G'' loss modulus, BCI blood clotting index, NIH National Institutes of Health

To enhance the injectability of 2% (w/v) Alg-AEMA hydrogel, 2% (w/v) polyvinylpyrrolidone, 1% (w/v) sodium carboxymethyl cellulose and 1% (w/v) glycerol were mixed as viscosity modifiers and the composite hydrogel was named AEC (Fig. 1g). Alg-AEMA hydrogel initiated by Eosin Y/NPG (AE) was used as a control in the following. Figure 1h presents the gelling transition of AEC under the 90 s illumination of a white light emitting diode (100 mW/cm^2^). Irradiation less than 70 s was insufficient for full photopolymerization of the hydrogel. Scanning electron microscope images showed that AEC exhibited highly porous network structures (Fig. 1i). Figure 1j showed a shear thinning behavior of AE and AEC, while AEC exhibited higher viscosity at the same shear rate. Dynamic time-sweep test and dynamic frequency-sweep test showed a rapid gelation process of AEC and its semi-solid state with the assistance of viscosity modifiers (Fig. 1k, l and Additional file 1: Fig. S2a). AEC exhibited better injectability (Fig. 1m, Additional file 2: Movie S1) and stability before photopolymerization (Additional file 1: Fig. S2b) due to the addition of the modifiers. Compression test showed that although the maximum compressive strength decreased [(6.36 ± 1.15) kPa vs. (16.30 ± 0.87) kPa] after the addition of modifiers, there were no significant differences between the compressive modulus of AEC and AE [(239.84 ± 55.25) Pa vs. (174.95 ± 56.25) Pa] (Fig. 1n, Additional file 1: Fig. S3a, b).

Furthermore, we tested the cytocompatibility, hemocompatibility and degradability to assess the biosafety of the composite hydrogel. Cell Counting Kit-8 assay (Fig. 1o) and fluorescence imaging of the Live/Dead staining (Additional file 1: Fig. S4a) showed high proportion of living cells after 72 h incubation with leaching solution of AEC. The photopolymerization process of AEC also exhibited good cytocompatibility (Additional file 1: Fig. S4b). In vitro hemolysis test showed the hydrogels exhibited superior hemocompatibility (Fig. 1p). AEC showed a better in vitro degradability (Additional file 1: Fig. S5). Subcutaneous implantation of the hydrogels did not cause obvious inflammation reaction and the porous structure of AEC enabled faster tissue cell migration and organization than AE over time (Fig. 1q, r).

Blood clotting index value and whole blood clotting time were generated as indicators for further investigation of in vitro coagulation function of AEC [4]. The lower blood clotting index values and relatively shorter blood clotting time of AEC than that of clinically used cellulose gauze and gelatin sponge suggested an increase of blood clotting ability in the hydrogel groups (Fig. 1s, t). Scanning electron microscope images in Fig. 1u and immunofluorescence staining of CD62P in Fig. 1v revealed that AEC can accelerate blood coagulation by stimulating the formation of fibrin network and activating platelets. Mouse liver trauma model was adopted for the measurement of in vivo hemostatic performance (Fig. 1w, x). AEC rapidly sealed the wound and terminated the mouse liver bleeding with total blood loss of (47.40 ± 7.61) mg and clotting time of (85.00 ± 3.16) s, which were dramatically lower than those in the gauze or sponge treated groups (Fig. 1y, z and Additional file 3: Movie S2). Factors affecting the hemostatic efficiency of alginate compound hydrogel were mainly proportion of hydrogel components, intensity of light source, crosslinking time, etc. For instance, lower concentration of Alg-AEMA cannot form hydrogels with sufficient mechanical strength. Insufficient light source intensity will prolong the photocuring time and insufficient photopolymerization can affect the hemostatic effect due to the inability to firmly adhere to the bleeding point. Although increasing the amount of photoinitiators can accelerate the photopolymerization, the potential cytotoxicity of the photoreaction system needs to be considered. Therefore, AEC can be considered as an injectable hydrogel with both good hemostatic function and good biological safety.

In summary, our study provided a new photoinitiator system Eosin Y/NPG, which was currently the most efficient white light photoinitiator commonly used, and an injectable photo-crosslinkable hydrogel AEC with good biocompability, which was proved to be a promising strategy for rapid hemorrhage control.

### Supplementary Information

**Additional file 1**. Materials and Methods. **Table S1** The formulas of eleven resins used in photopolymerization kinetic studies. **Table S2** The formulas of the hydrogels used in this study. **Fig. S1** Evaluation of cytotoxicity of white light and near-UV light. **Fig. S2** Rheological property of the hydrogels. **Fig. S3** Compression test of the hydrogels. **Fig. S4** Cytocompatibility of the hydrogels. **Fig. S5** In vitro degradation curves of AE and AEC (*n* = 3).

**Additional file 2**. **Movie 1** Injectability of AE and AEC.

**Additional file 3**. **Movie 2** Hemostatic performance in mouse liver trauma model.

## Data Availability

The data and materials used in the current study are all available from the corresponding author upon reasonable request.

## Abbreviations

AE: Alg-AEMA hydrogel initiated by Eosin Y/NPG
AEC: Alg-AEMA-based Eosin Y/NPG-initiated composite hydrogel
Alg-AEMA: Methacrylate alginate
DBC: Double bond conversion
NPG: N-phenylglycine
NVP: N-vinyrrolidone
PRP: Platelet-rich plasma
Ru: Tris-bipyridyl ruthenium hexahydrate
SPS: Sodium persulfate
