# Supplementary material for: White-light-induced synthesis of injectable alginate-based composite hydrogels for rapid hemostasis
Source: Mil Med Res. 2023 Oct 17;10:47. doi: 10.1186/s40779-023-00483-7 (PMC10580567; doi:10.1186/s40779-023-00483-7)
Supplement: Supplementary file 1 — Additional file 1. Materials and Methods. Table S1 The formulas of eleven resins used in photopolymerization kinetic studies. Table S2 The formulas of the hydrogels used in this study. Fig. S1 Evaluation of cytotoxicity of white light and near-UV light. Fig. S2 Rheological property of the hydrogels. Fig. S3 Compression test of the hydrogels. Fig. S4 Cytocompatibility of the hydrogels. Fig. S5 In vitro degradation curves of AE and AEC (n = 3). [file 40779_2023_483_MOESM1_ESM.docx]

**Materials and methods**

**Materials**

Methacrylate alginate (Alg-AEMA) was synthesized according to standard experimental steps. Eosin Y, polyvinylpyrrolidone, sodium carboxymethyl cellulose, glycerol and tris-bipyridyl ruthenium hexahydrate were purchased from Sigma-Aldrich (Shanghai, China). N-phenylglycine (NPG) was purchased from Alfa Aesar (Shanghai, China). Sodium persulfate, triethanolamine, N-vinyrrolidone and poly(ethylene glycol) diacrylate 400 (PEGDA400) were purchased from Aladdin (Shanghai, China).

**Photopolymerization kinetic study**

Eleven resins (R1 – R11) listed in **Additional file 1: Table S1** were used to study the photoinitiation properties of Eosin Y/NPG, in which two visible light initiator systems (tris-bipyridyl ruthenium hexahydrate/sodium persulfate, Eosin Y/triethanolamine/N-vinyrrolidone reported elsewhere were employed as references. The monomer PEGDA400, photoinitiators and other additives (solvent and/or coinitiator) were mixed well and injected into a homemade glass cell with a thickness of 0.3 mm for measurement. The real-time near-infrared spectrum of each resin was recorded under irradiation with a white light light-emitting diode (100 mW/cm^2^) and the double bond conversion of PEGDA400 was calculated from the change in the C = C absorption peak area within (6100 – 6250)/cm.

**Preparation of the hydrogels**

The Alg-AEMA hydrogel and the composite hydrogel were prepared according to **Additional file 1: Table S2**. Take Alg-AEMA-based Eosin Y/NPG-initiated composite hydrogel as an example, 1 mg Eosin Y, 10 mg NPG and 0.2 g Alg-AEMA were dissolved in 5 ml ultrapure water. Then 0.2 g polyvinylpyrrolidone, 0.1 g sodium carboxymethyl cellulose and 0.1 ml glycerol were added into 5 ml ultrapure water to obtain viscosity modifier. The two parts were fully stirred and evenly mixed to obtain the composite hydrogel. Alg-AEMA cyrogel was sterilized via ultraviolet irradiation, and the hydrogel solution was prepared under sterile condition. The hydrogel solution was sealed and stored at 4 ℃ for subsequent experiments.

**Scanning electron microscope (SEM) imaging**

The morphology of the materials was characterized with a SEM (HITACHI S-4300). Each sample (photopolymerized for 90 s) was placed in ultrapure water until swelling equilibrium. Then each was cut into cubes and the cross-section was used for characterization. Each sample was lyophilized and mounted on specimen holders with carbon adhesive tape. Each sample was sputtered with Au before imaging.

**Measurement of** **rheological properties**

The flow sweep test, oscillatory amplitude sweep and oscillation frequency test were used to evaluate the rheological property of the hydrogels by employing a rotational rheometer (ARES G2, TA). A 40 mm cone plate was used and the working gap was set at 1 mm. All tests were performed at room temperature. In flow sweep test, shear rate range of 0.1 s^-1^ to 100 s^-1^ was performed. In oscillatory amplitude sweep test, the frequency was kept at 1 rad/s, and the change of G′ and G′′ with amplitude strain were monitored. In oscillation frequency test, the strain was kept constant at 1%.

**Measurement of** **mechanical properties**

The compression test was used to evaluate the mechanical performance of the hydrogel by employing an electromechanical test system (Model E42, MTS) at room temperature. The compression speed was set to 1 mm/min. A cylindrical mold with an inner diameter of 13.8 mm was used for sample photopolymerization before compression test. The compressive modulus was calculated from the slope of the linear region of the stress-strain curve, and compressive modulus at 10% strain was selected for comparison. Breaking stress was the maximum stress at the break point. Each test was repeated three times.

**Blood collection and blood cells isolation**

All the animal experiments were performed according to the guidelines established by Institutional Animal Care and Use Committee of Chinese PLA General Hospital (S2020-407-01). All experimental animals were purchased from the SPF Biotechnology Limited. Mouse blood was collected under anesthesia according to a standard cardiac blood collection method. Sodium citrate (3.2%) single-use evacuated tubes were used for blood collection (blood/anticoagulant = 9/1). Anticoagulant whole blood was centrifuged at 100 g for 15 min to obtain platelet-rich plasma (PRP) and red blood cell suspension.

**Cytocompatibility of the hydrogels**

The cytocompatibility of photoinitiators or hydrogels was evaluated by following the ISO 10993-5:2009 with minor modification. Specifically, direct interaction assay and the leachable substances assay were used to evaluate the cytotoxicity of photoinitiators and hydrogels, respectively. National Institutes of Health/3T3 cells were cultured in Dulbecco’s modified eagle medium (DMEM) containing 10% fetal bovine serum and 1% penicillin-streptomycin. For direct contact assay, cells were incubated with complete DMEM with gradient concentration of NPG or Eosin Y for 24 h. For the leaching pattern, DMEM-based extracts of the hydrogels were added to each well for 24, 48 and 72 h. Cell counting kit-8 (CK04, Dojindo) assay, where optical density values at 450 nm (OD_450 nm_) were measured and relative cell viability was calculated, and Live/Dead staining (L3224, Invitrogen), where living cells were stained by calcein acetoxymethyl ester (green) and dead cells by ethidium homodimer-1 (red), were employed to evaluate the cytotoxicity according to standard methods. To value the impact of photopolymerization on cells, different amounts of hydrogels were injected and photo-crosslinked in the cell culture plate. The relative cell viability after 24 h of cocultivation was evaluated via cell counting kit-8 assay. Each test was repeated three times.

**Hemocompatibility of the hydrogels**

Hemolytic activity was evaluated via spectrophotometry. Briefly, 10 mg sample was dispersed in 1 ml pre-warmed normal saline in each tube before the addition of 20 μl red blood cell suspension. After the incubation at 37 ℃ for 1 h, the optical density value at 540 nm (OD_540 nm_) of the supernatant was read by a multimode microplate reader (Spark 10M, Tecan). Triton X-100 (35501-15, Nacalai) was used as a positive control. Each test was repeated three times.

**In vitro and in vivo degradation**

For in vitro degradation test, each sample was weighed immediately after crosslinking and placed in a petri dish at room temperature. Each petri dish was filled with 10 ml ultrapure water which was changed every day during the experiment. The degradation percentage of each sample was calculated as (W_s_ - W_t_)/ W_s_ × 100%, where W_s_ was the weight at swelling equilibrium point, and W_t_ was the weight at pre-set time t. Each test was repeated three times.

For in vivo biodegradation test, sterilized crosslinked columnar samples (10 mm-diameter and 2 mm-height) were subcutaneously implanted in 8-week-old C57BL/6 mice for 1, 3 and 5 weeks. The mice were sacrificed at pre-set time and the samples were excised with the adjacent tissues. Paraffin sectioning and hematoxylin-eosin staining were performed according to standard procedures.

**Measurement of in vitro pro-coagulant ability**

Blood clotting index (BCI) and whole blood clotting assay were employed to evaluate the pro-coagulant ability in vitro. For BCI test, pre-warmed samples were placed in Eppendorf tubes, and 50 μl citrated whole blood was dropped to each tube. After incubation for 5 and 10 min at 37 °C, 2 ml ultrapure water was added into each tube, and OD_540 nm_ of the supernatant (OD_sample_) was determined by a multimode reader. The tube without sample was used as a negative control and its OD_540 nm_ was called as OD_control_. The experiment process was recorded by a digital camera. The BCI was calculated according to the following equation: BCI (%) = OD_sample_/OD_control_ × 100 %.

For whole blood clotting time assay, 500 μl anticoagulant blood was added to each tube with 50 mg preheated sample. The time for whole blood clotting was recorded immediately after the addition of 50 μl of 0.2 mol/L calcium chloride. Photographs during the blood-clotting process were recorded by a digital camera. Each test was repeated three times.

For SEM imaging of the blood clot, each sample was fixed in 2% glutaraldehyde for 12 h and gradient-dehydrated in ethanol before lyophilized.

**Measurement of in vivo hemostatic ability**

Mouse (male C57BL/6, 22 – 25 g) liver trauma model was used to test the hemostatic effect in vivo in this research. The experimental animals were randomly divided into different groups of different hemostatic treatments. First, mouse was anesthetized by pentobarbital. Then, the liver was dissected and the lobe was gently lifted outside the abdominal cavity. Pre-weighed filter paper lower lining with a water barrier was placed beneath the liver lobe. A 1.6 mm-diameter syringe needle was used to penetrate the liver and the hydrogel was immediately applied and crosslinked at the bleeding site. The weight of the filter paper with blood was measured, and the mass of blood was calculated. The clotting time in vivo was recorded. In this study, the blood loss of each group was less than 10% of the total blood volume, thus the experimental animals under anesthesia would not be life-threatening. The experimental animals were euthanized by excessive anesthesia after the experiment. Each test was repeated five times.

**Immunofluorescence staining**

The activation of platelets was evaluated by immunofluorescence staining for CD62p. After incubation with different samples for 5 min, PRP was made into PRP smear and fixed with 1% paraformaldehyde. Rabbit polyclonal anti-SELP (A01241-1, Boster) and CoraLite594–conjugated goat anti-rabbit IgG (SA00013-4, Proteintech) were used in 1:100 and 1:200, respectively. Images were observed with a laser confocal scanning microscope (Leica).

**Statistical analysis**

All values are presented as the means ± standard deviation calculated from the average of at least three biological replicates. Statistical analysis was performed in GraphPad Prism 8.0. Comparisons were analyzed with one-way analysis of variance (ANOVA) (^*^*P* < 0.05, ^**^*P* < 0.01, ^***^*P* < 0.001, ^****^*P* < 0.0001). Curve fitting of compression test was performed in OriginLab Origin 2021b.

**Table S1** The formulas of eleven resins used in photopolymerization kinetic studies

| **Resin** | **Eosin Y (mg)** | **H_2_O (g)** | **PEGDA (g)** | **TEOA (mg)** | **NVP (mg)** | **Ru (mg)** | **SPS (mg)** | **NPG (mg)** |
| --- | --- | --- | --- | --- | --- | --- | --- | --- |
| R1 | 1.0 | 1 | 10.0 | 0 | 0 | 0 | 0 | 1.0 |
| R2 | 1.0 | 1.0 | 10.0 | 0 | 0 | 0 | 0 | 2.0 |
| R3 | 1.0 | 1.0 | 10.0 | 0 | 0 | 0 | 0 | 3.0 |
| R4 | 1.0 | 1.0 | 10.0 | 0 | 0 | 0 | 0 | 5.0 |
| R5 | 1.0 | 1.0 | 10.0 | 0 | 0 | 0 | 0 | 10.0 |
| R6 | 1.0 | 1.0 | 10.0 | 0 | 0 | 0 | 0 | 10.0 |
| R7 | 2.0 | 1.0 | 10.0 | 0 | 0 | 0 | 0 | 10.0 |
| R8 | 3.0 | 1.0 | 10.0 | 0 | 0 | 0 | 0 | 10.0 |
| R9 | 1.0 | 1.0 | 10.0 | 0 | 0 | 0 | 0 | 10.0 |
| R10 | 1.0 | 1.0 | 10.0 | 10.0 | 10.0 | 0 | 0 | 0 |
| R11 | 0 | 1.0 | 10.0 | 0 | 0 | 1.0 | 10.0 | 0 |

*TEOA* triethanolamine, *NVP* N-vinyrrolidone, *Ru* tris-bipyridyl ruthenium hexahydrate, *SPS* sodium persulfate, *NPG* N-phenylglycine, *PEGDA* poly(ethylene glycol) diacrylate

**Table S2** The formulas of the hydrogels used in this study

| **Sample** | **Alg-AEMA(g/L)** | **Eosin Y (g/L)** | **NPG (g/L)** | **PVP (g/L)** | **CMC (g/L)** | **Glycerol (ml/L)** |
| --- | --- | --- | --- | --- | --- | --- |
| AE | 20.0 | 0.1 | 1.0 | 0 | 0 | 0 |
| AEC | 20.0 | 0.1 | 1.0 | 20.0 | 10.0 | 10.0 |

*NPG* N-phenylglycine, *PVP* polyvinylpyrrolidone, *CMC* sodium carboxymethyl cellulose, *Alg-AEMA* methacrylate alginate, *AE* Alg-AEMA hydrogel initiated by Eosin Y/NPG, *AEC* Alg-AEMA-based Eosin Y/NPG-initiated composite hydrogel

**
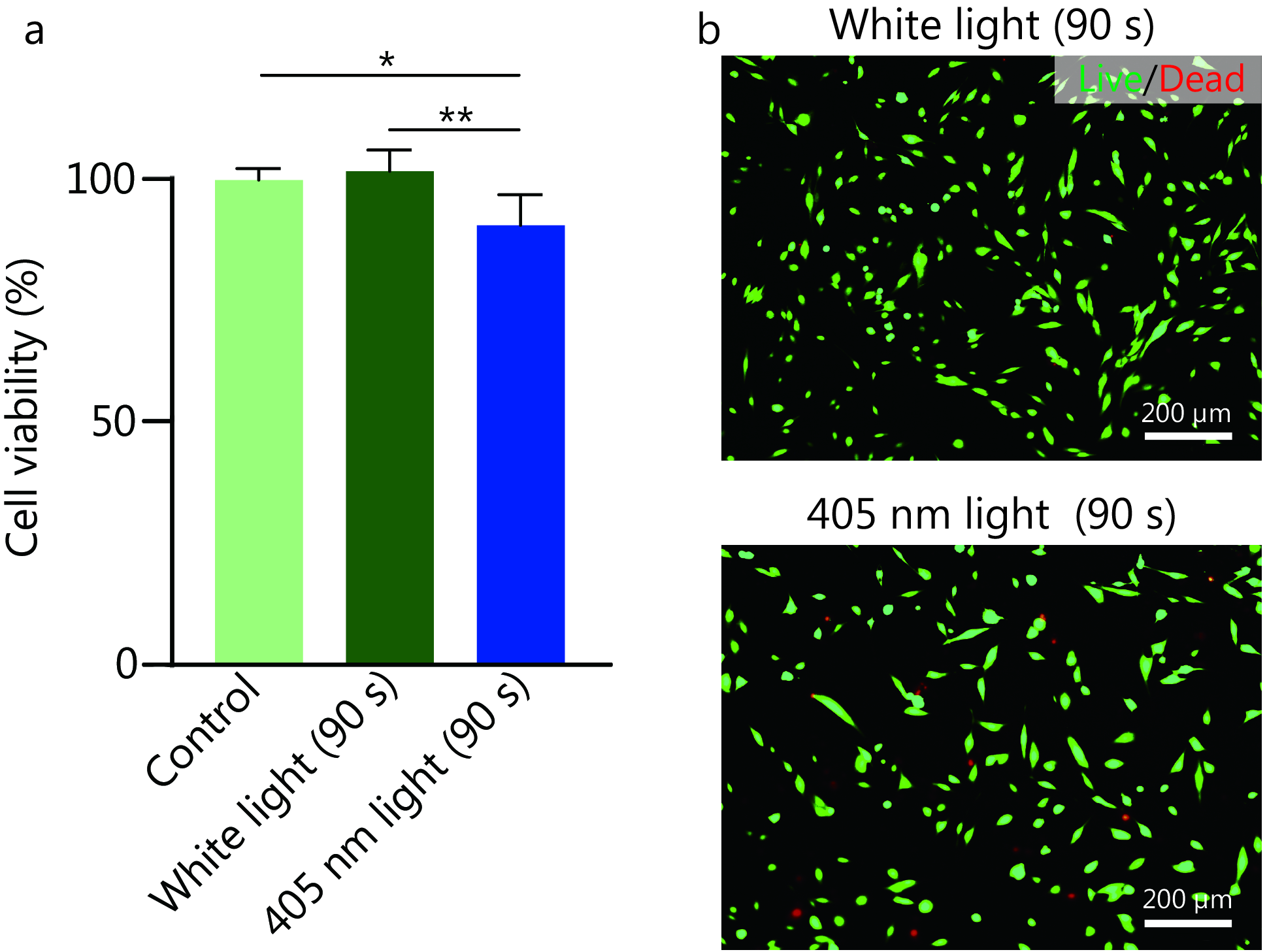
**

**Fig. S****1** Evaluation of cytotoxicity of white light and near-UV light. Relative cell viability (**a**) and fluorescent images of Live/Dead staining (**b**) of NIH/3T3 fibroblasts after white-light/near-UV-light irradiation for 90 s and incubation for 24 h. Scale bar = 200 μm. All statistical data are represented as mean ± SD (*n* = 3, ^*^*P* < 0.05, ^**^*P* < 0.01, One-Way ANOVA, Tukey’s post hoc test). UV ultraviolet, NIH National Institutes of Health

**
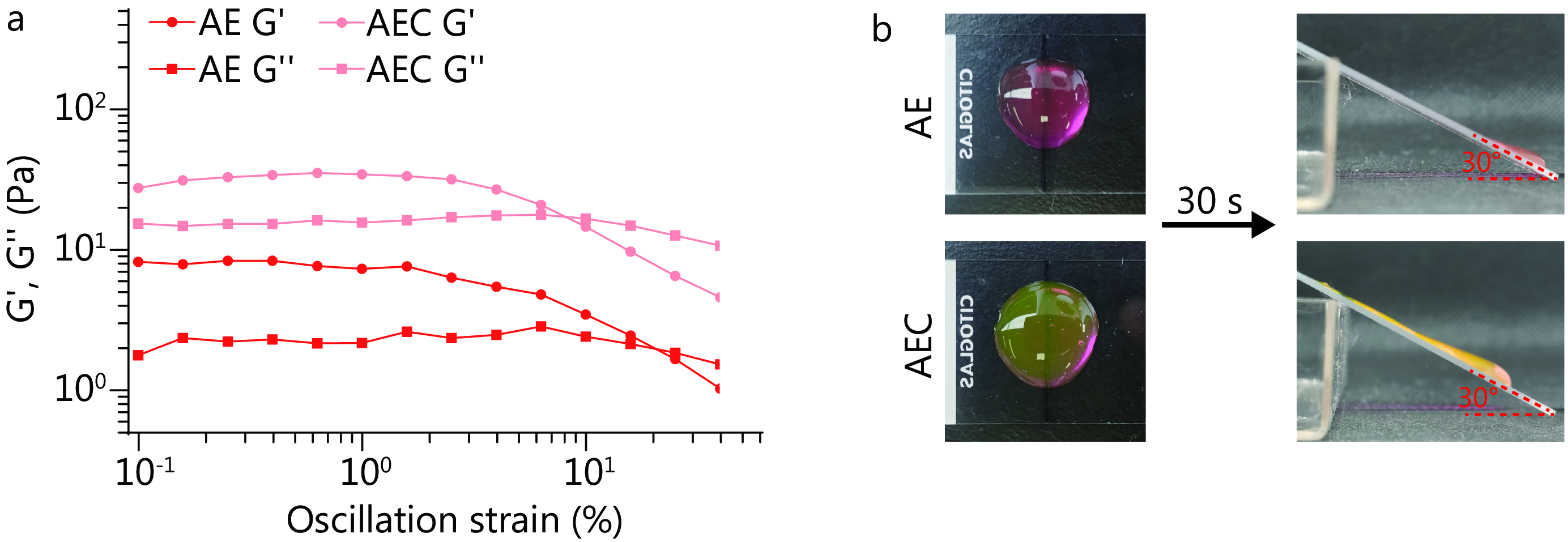
**

**Fig. S2** Rheological property of the hydrogels. **a** G′ and G′′ of AE and AEC on strain amplitude sweep at a fixed angular frequency (1 rad/s). **b** Macroscopic observation showing the viscosity performance of the hydrogel. AE Alg-AEMA hydrogel initiated by Eosin Y/NPG, AEC Alg-AEMA-based Eosin Y/NPG-initiated composite hydrogel, NPG N-phenylglycine, G' storage modulus, G'' loss modulus


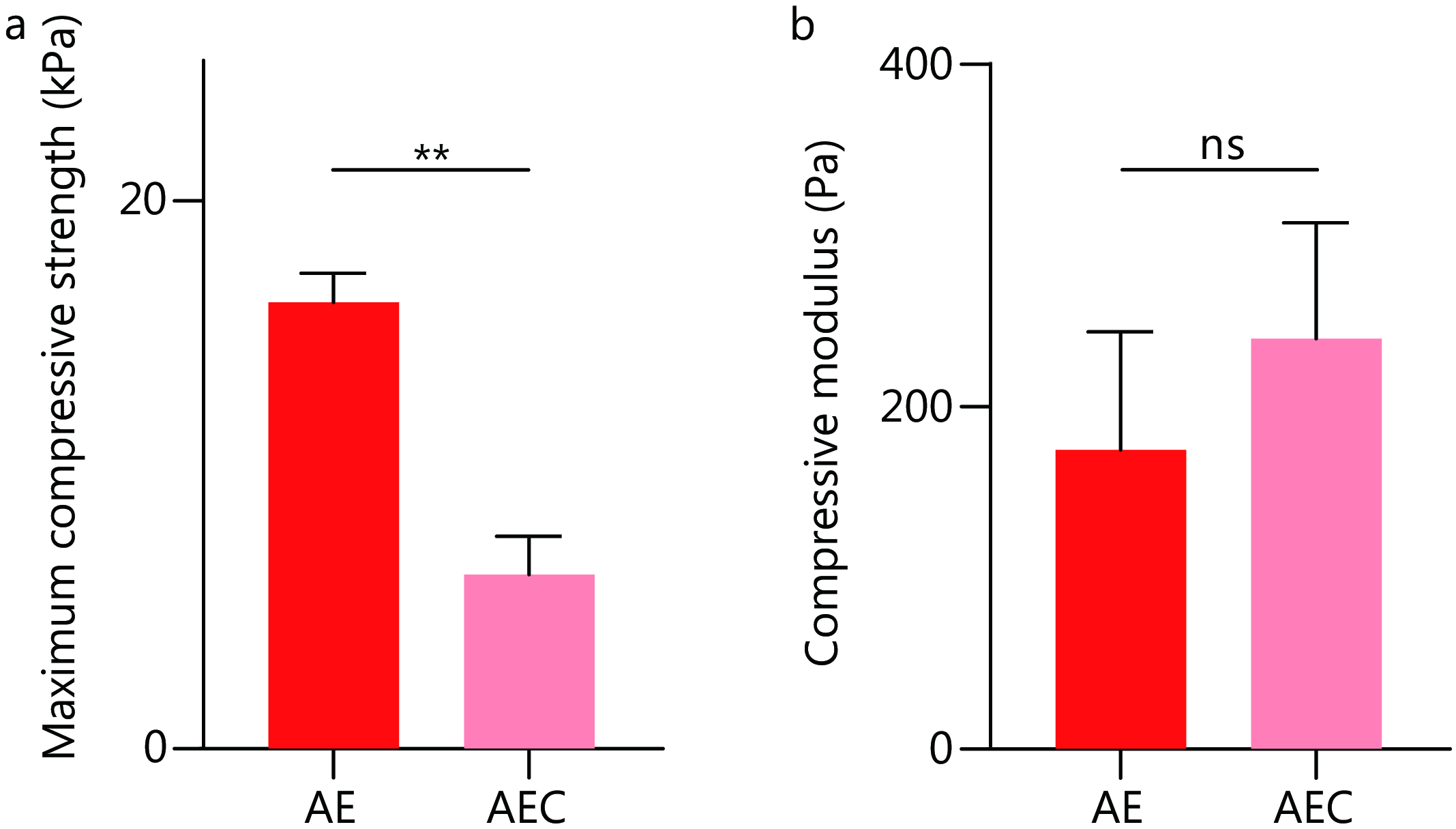


**Fig. S3** Compression test of the hydrogels. The observed maximum compressive strength (**a**) and compressive modulus (**b**, at 10% compressive strain) of each sample. Exposure time was 120 s. All statistical data are represented as mean ± SD (*n* = 3, ^**^*P* < 0.01, One-Way ANOVA, Tukey’s post hoc test). AE Alg-AEMA hydrogel initiated by Eosin Y/NPG, AEC Alg-AEMA-based Eosin Y/NPG-initiated composite hydrogel, NPG N-phenylglycine


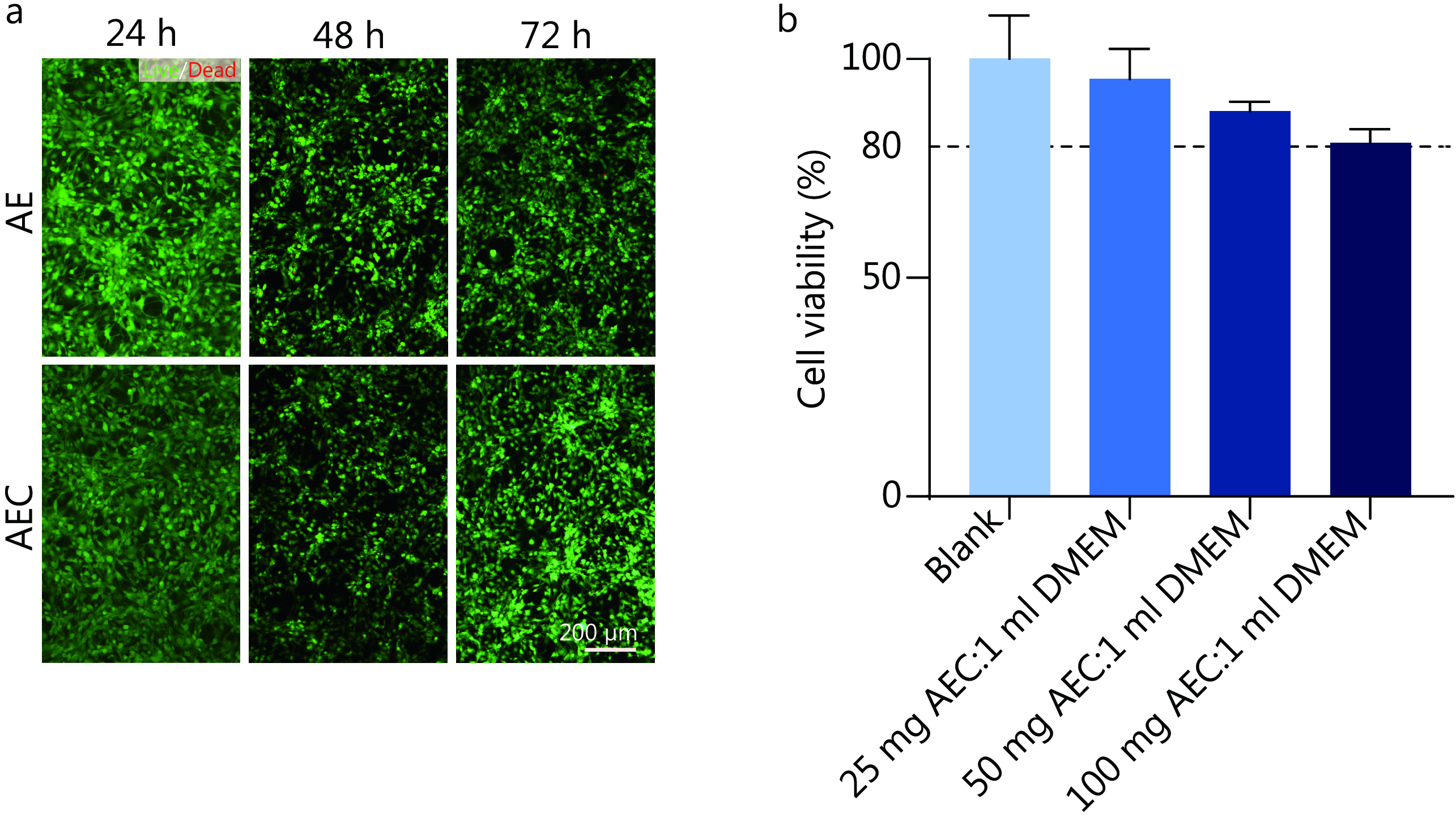


**Fig. S4** Cytocompatibility of the hydrogels. **a** Live/dead staining fluorescent imaging of NIH/3T3 fibroblasts after incubation with hydrogel extract for 24, 48 and 72 h. Scale bar = 200 μm. **b** Relative cell viability during photopolymerization of AEC (*n* = 3). AE Alg-AEMA hydrogel initiated by Eosin Y/NPG, AEC Alg-AEMA-based Eosin Y/NPG-initiated composite hydrogel, DMEM dulbecco’s modified eagle medium, NPG N-phenylglycine, NIH National Institutes of Health


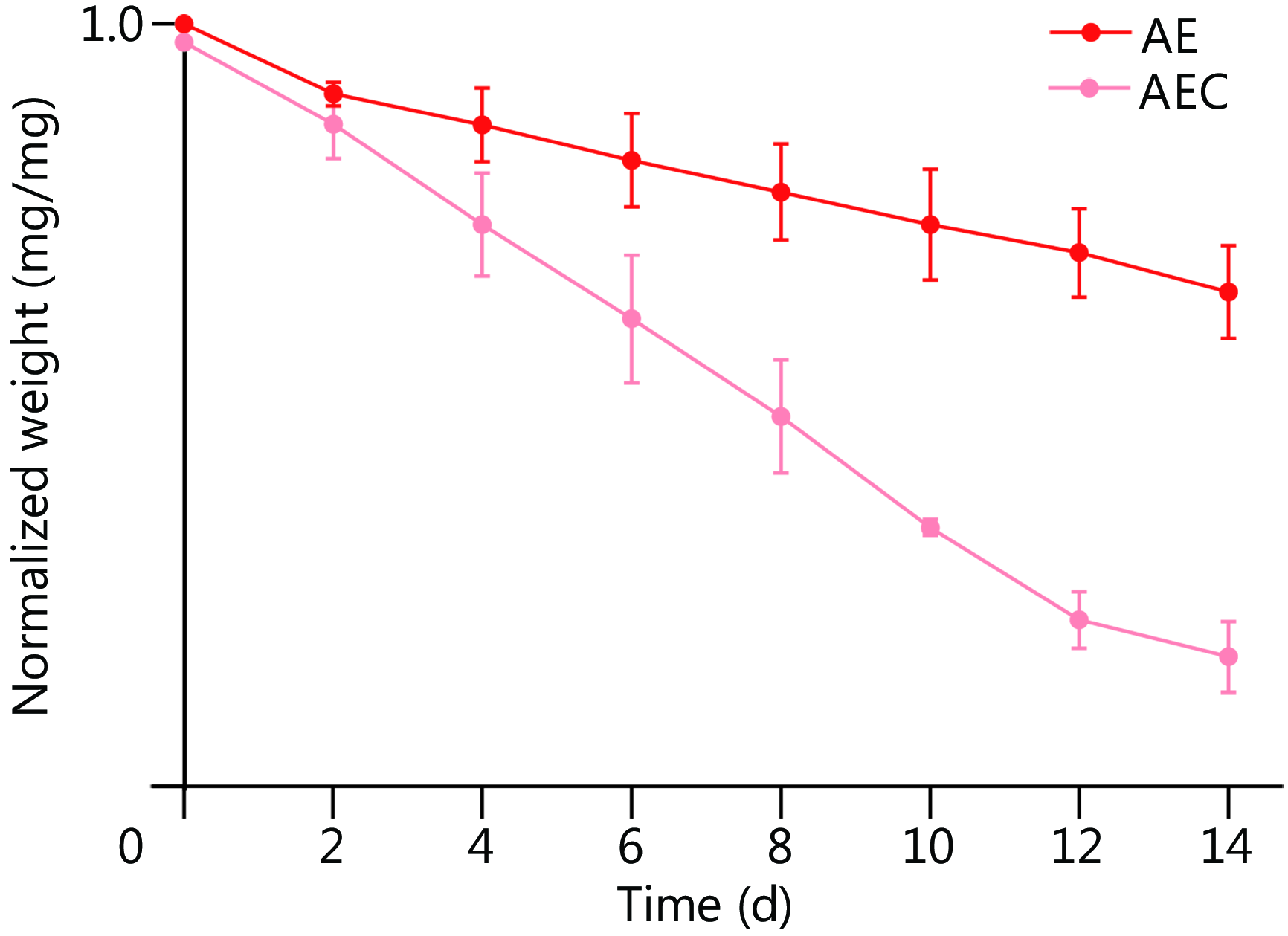


**Fig. S5** In vitro degradation curves of AE and AEC (*n* = 3). AE Alg-AEMA hydrogel initiated by Eosin Y/NPG, AEC Alg-AEMA-based Eosin Y/NPG-initiated composite hydrogel, NPG N-phenylglycine
